# Therapeutic effects of micro-RNAs in preclinical studies of acute kidney injury: a systematic review and meta-analysis

**DOI:** 10.1038/s41598-021-88746-y

**Published:** 2021-04-27

**Authors:** Sarah Zankar, Mayra Trentin-Sonoda, Jose L. Viñas, Rosendo A. Rodriguez, Adrian Bailey, David Allan, Kevin D. Burns

**Affiliations:** 1 Department of Medicine, The Ottawa Hospital and University of Ottawa, 501 Smyth Road, Ottawa, ON K1H 8L6 Canada; 2 Division of Nephrology, Department of Medicine, Kidney Research Centre, Ottawa Hospital Research Institute, University of Ottawa, 1967 Riverside Drive, Rm. 535, Ottawa, ON K1H 7W9 Canada

**Keywords:** Pathogenesis, Nephrology, Kidney, Kidney diseases, Experimental models of disease, Preclinical research

## Abstract

AKI has a high mortality rate, may lead to chronic kidney disease, and effective therapies are lacking. Micro-RNAs (miRNAs) regulate biologic processes by potently inhibiting protein expression, and pre-clinical studies have explored their roles in AKI. We conducted a systematic review and meta-analysis of miRNAs as therapeutics in pre-clinical AKI. Study screening, data extraction, and quality assessments were performed by 2 independent reviewers. Seventy studies involving 42 miRNA species were included in the analysis. All studies demonstrated significant effects of the miRNA intervention on kidney function and/or histology, with most implicating apoptosis and phosphatase and tensin homolog (PTEN) signaling. Fourteen studies (20.0%) examined the effect of miRNA-21 in AKI, and meta-analysis demonstrated significant increases in serum creatinine and kidney injury scores with miR-21 antagonism and pre-conditioning. No studies reported on adverse effects of miRNA therapy. Limitations also included lack of model diversity (100% rodents, 61.4% ischemia–reperfusion injury), and predominance of male sex (78.6%). Most studies had an unclear risk of bias, and the majority of miRNA-21 studies were conducted by a single team of investigators. In summary, several miRNAs target kidney function and apoptosis in pre-clinical AKI models, with data suggesting that miRNA-21 may mediate protection and kidney repair.

Systematic review registration ID: CRD42019128854.

## Introduction

Acute kidney injury (AKI) is a worldwide public health issue, estimated to result in 1.7 million deaths annually^1^. During hospital admission, approximately 20% of adult patients develop AKI, with a prevalence in the critically ill approaching 50%^2–4^. A recent meta-analysis of 82 studies involving more than 2 million hospitalized adults for 1 year demonstrated that patients with AKI have a threefold increased risk of developing new or progressive chronic kidney disease (CKD), an almost fourfold increase in kidney failure, and a twofold increase in mortality rates^5^.

Despite recent advances in understanding the pathogenesis of AKI, there are no established treatments to accelerate kidney recovery and repair in humans. Micro-RNAs (miRNAs) are a class of small noncoding single stranded RNA molecules that are involved in the regulation of multiple pathophysiological processes, including oxidative stress, metabolic disorders, mitochondrial function and tissue injury^6–8^. miRNAs primarily target non-coding 3′ untranslated regions (UTRs) of mRNAs leading to inhibition of translation or mRNA degradation, and are involved in protective or pathogenic molecular pathways in experimental models^9^. Although miRNAs have important roles as biomarkers of disease severity in AKI, pre-clinical studies have investigated their potential as therapeutic agents^10–13^. In this regard, miRNAs can act pathogenically and promote kidney inflammation, apoptosis and fibrosis in AKI, or exert anti-inflammatory, anti-apoptotic or even pro-angiogenic effects, protecting against kidney injury^14–16^. Despite widespread interest in miRNA therapeutics, however, the effect of miRNA administration in pre-clinical models of AKI remains unclear. This is particularly relevant since clinical trials in humans are currently underway assessing the safety and efficacy of miRNA therapy in cancer, hepatitis C, cardiac disease, and other conditions^17^***.***

The primary aim of this study was to conduct a systematic review of studies involving effects of miRNA interventions in pre-clinical AKI models. A secondary predefined objective was to determine potential adverse effects of miRNA administration in pre-clinical AKI^18^. Our results identified potential miRNA candidates for AKI therapy, gaps in current knowledge, and barriers to translation for human application.

## Results

### Study characteristics

**Figure 1:**
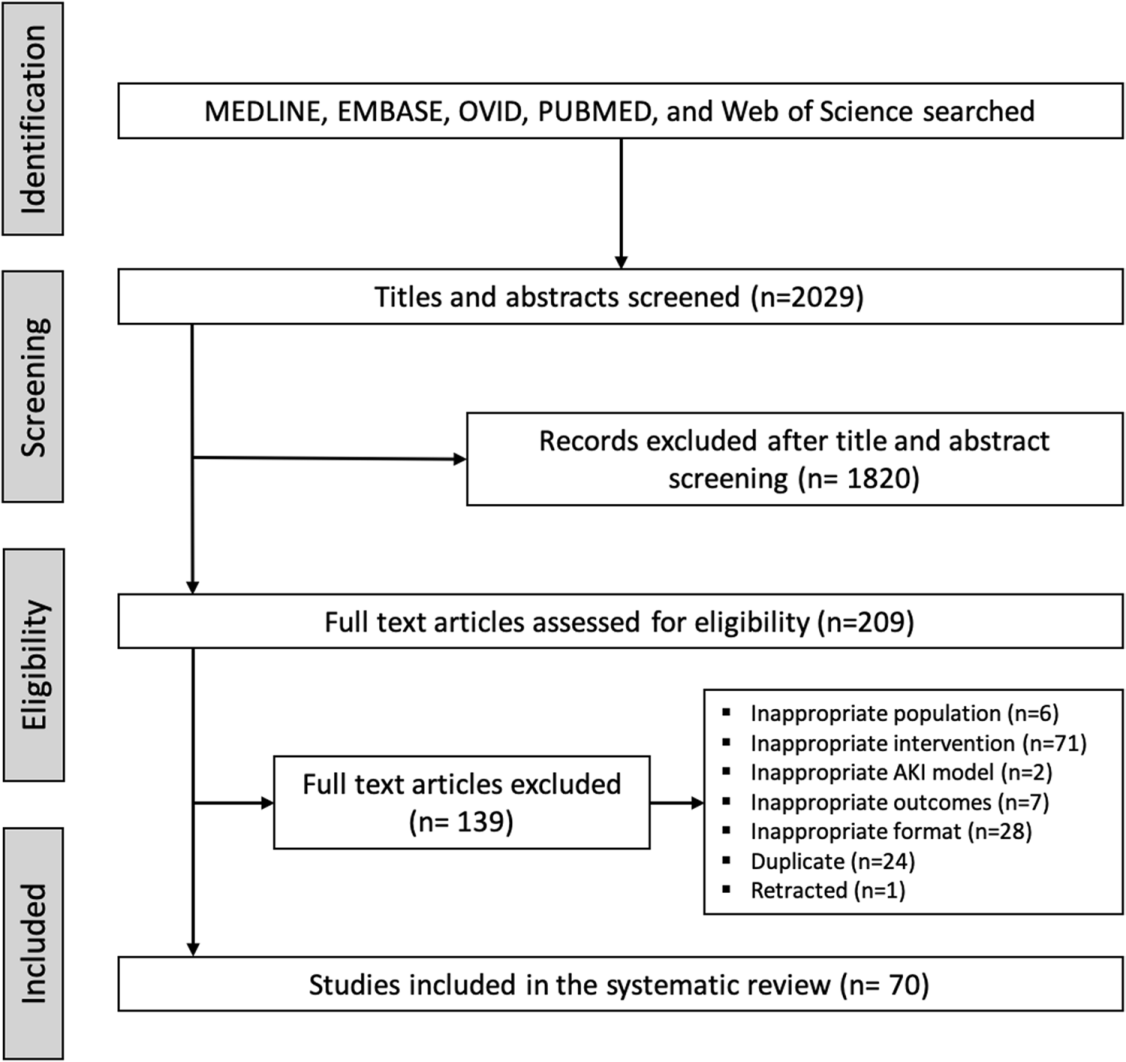
PRISMA flowchart. Number of titles, abstracts and full texts screened, resulting in 70 studies included in the qualitative synthesis. AKI = Acute kidney injury.

The search identified 2029 titles and abstracts (Fig. 1). After initial screening, 1820 reports were excluded. The remaining 209 manuscripts were subject to full-text review and abstraction, and 70 manuscripts met inclusion criteria. Supplementary Table S1 depicts the detailed features of these studies. The geographic location of the corresponding authors’ institutional affiliations demonstrated a predominance of studies in Asia (51 studies, 72.9%) followed by Europe (9 studies, 12.9%), North America (8 studies, 11.4%) and South America (2 studies, 2.9%).

**Figure 2:**
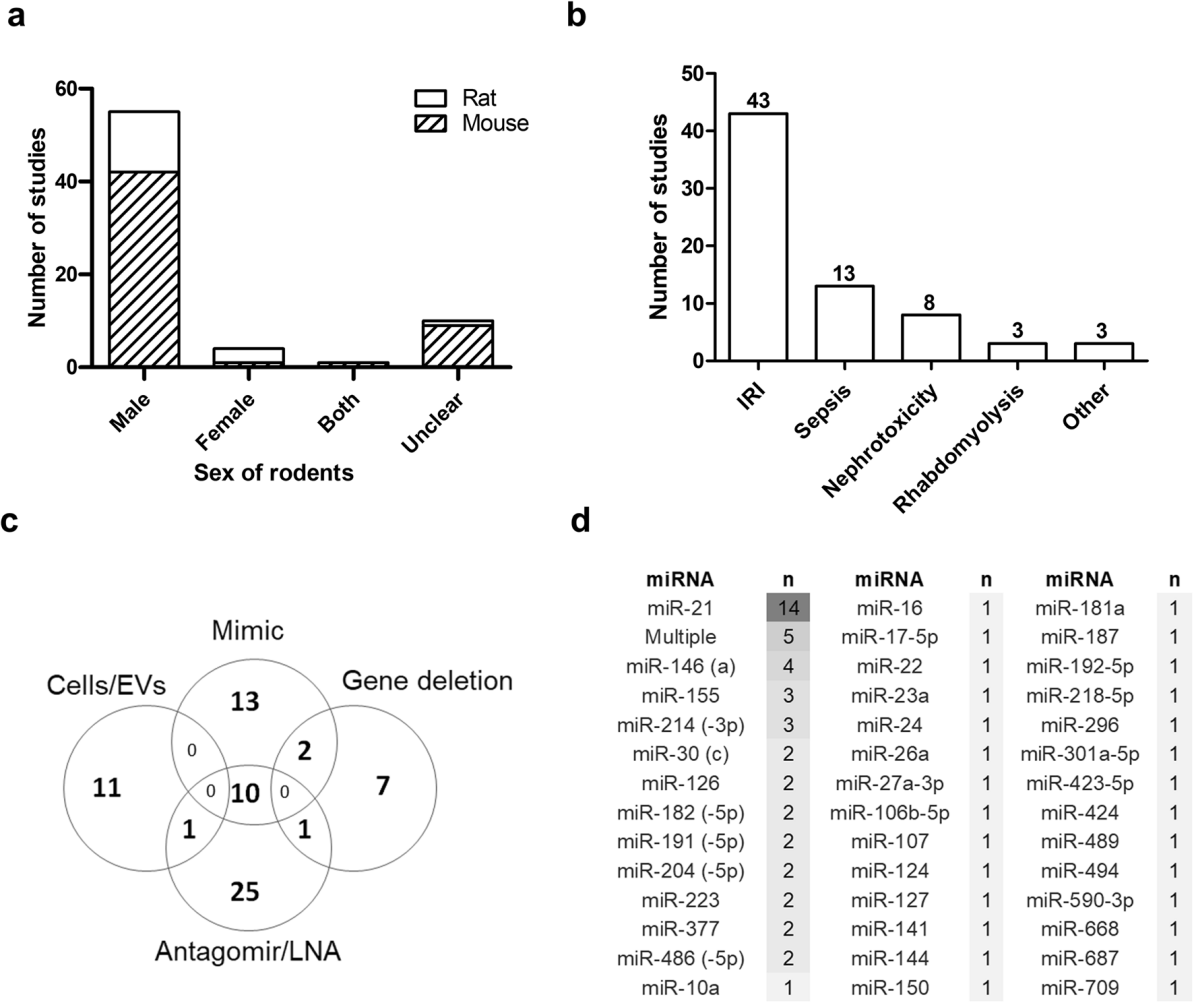
Study characteristics. Distribution of studies by (**a**) species and sex of rodents, (**b**) AKI model (IRI, ischemia–reperfusion injury), (**c**) Types of interventions used in the included studies (EV, extracellular vesicle; LNA, locked nucleic acid), and (**d**) Number of studies (n) per miRNA; further definition of miRNAs provided in some studies is designated in parenthesis.

All studies were conducted in rodents (75.7% mice, 24.3% rat), and the majority (78.6%) included males only. Both sexes were studied in only 1.4% of the reports, and 14.3% did not disclose animal sex (Fig. 2a). The leading AKI models included ischemia–reperfusion injury (IRI) (61.4%), sepsis (18.6%), and nephrotoxicity (11.4%) (Fig. 2b). Ten (14.3%) studies involved a co-intervention of either ischemic, xenon, cobalt chloride or lipopolysaccharides preconditioning before the miRNA intervention.

Direct miRNA administration alone occurred in 58 studies (82.9%), with delivery of miRNA mimic alone in 13 (18.6%), antagomirs alone in 25 (37.1%) and use of both in 10 (14.3%). Ten studies (14.3%) used a gene deletion model with AKI. A minority of studies (11, 15.7%) used only an indirect miRNA intervention by administration of cells, extracellular vesicles, or exosomes, with 7 of these studies involving overexpression of miRNA agomirs or antagomirs, and 4 studies involving use of ribonuclease (RNAse) to eliminate miRNAs. One study used both a direct (antagomir) and indirect (exosome) interventions (Fig. 2c).

The effects of 42 distinct miRNA variants were evaluated, with most studies focused on miRNA-21 (n = 14), followed by miRNA-146a (n = 4) and miRNA-155 (n = 3) (Fig. 2d). Delivery of miRNA mimics/antagomirs occurred mainly by intravenous (i.v.) route (65.7%), while intraperitoneal (i.p.) injection was used in 11.4%. Administration of miRNAs occurred prior to AKI in a majority of studies (68.6%), with 15.7% at AKI induction, and 10.0% post-AKI. Timing of administration was unclear in 5.7%.

Serum Cr was evaluated as an outcome measure in 81.4% of studies, while 67.1% measured BUN or urea. 56.3% of studies reported kidney histologic injury scores, 43.7% (31) evaluated apoptosis, and a minority (12.7%) reported inflammatory cell infiltration or kidney fibrosis (5.6%).

All 70 studies (100%) demonstrated a significant effect of the miRNA intervention on kidney function and/or structure, with most interventions (75.7%) having a protective effect.

### Effect of miRNA intervention and model

Table 1 depicts studies stratified according to intervention (miRNA mimic, antagomir, or gene deletion), and most frequent AKI models (IRI or sepsis), along with the impact of intervention and model on changes in serum Cr as outcome measure (insufficient numbers of studies reported kidney injury scores to conduct a comparative analysis with this outcome measure). For this analysis, we focused on studies conducted in mice, which represented the majority (86.0%). Pooled mean differences in serum Cr were compared according to intervention and model, along with the 95% confidence intervals (CIs). As shown in Table 1, there was a large variability in the directionality and magnitude of the individual effects within each of the 3 interventions (mimic [agomir], antagomir and gene deletion). Independent of injury model, 59% (13/22) of studies that used antagomir as intervention were associated with an increase in serum Cr levels, while mimic and gene deletion studies reported increased serum Cr in 33% and 40%, respectively. The group average of the individual mean differences versus controls and their 95% CIs for mimic, antagomir, and gene deletion studies were respectively − 21.2 µM (− 31.9, − 10.5), 8.1 µM (1.2, 15.0) and 4.2 µM (− 18.3, 26.8). Due to the high heterogeneity associated with different miRNA variants and injury models, we did not conduct a statistical analysis.

**Table 1:** Effect of intervention and model on serum creatinine in mice with AKI.

Study^+^	miRNA	AKI model	Comparator SCr (µM)	SD	n	Intervention SCr (µM)	SD	n	Mean difference	95% CI ( +)	95% CI (−)
**Mimic**											
Wang et al., (2020)^3^*	miR-218-5p	IRI	28.1	6.4	9	18.4	5.5	9	− 9.7	− 4.2	− 15.2
Zhao et al., (2020)^7^*	miR-27a-3p	IRI	125.1	34.8	3	162.7	60.4	3	37.6	116.4	− 41.3
Chen et al., (2019)^8^*	miR-424	IRI	217.5	24.5	NS	128.3	17.0	NS	− 89.2	− 65.4	− 113.0
Liu et al., (2019)^11^*	miR-377	IRI	209.2	44.0	8	229.8	99.8	8	20.6	96.2	− 55.0
Zhu et al., (2019)^21^*	miR-204-5p	IRI	62.3	8.6	6	28.6	4.5	6	− 33.7	− 25.9	− 41.5
Song et al., (2018)^29^*	multiple including miR-17–92 cluster	IRI	51.1	3.3	5	37.6	4.4	5	− 13.5	− 8.7	− 18.3
Wei et al., (2018)^30^*	miR-668	IRI	203.4	53.6	6	127.6	50.2	7	− 75.8	− 19.3	− 132.3
Hao et al., (2017)^37^*	miR-17-5p	IRI	209.0	31.6	5	130.2	24.0	5	− 78.8	− 44.0	− 113.6
Huang et al., (2018)^38^*	miR-146	IRI	59.0	8.2	6	40.8	2.0	6	− 18.2	− 11.4	− 25.0
Chen et al., (2016a)^49^*	miR-16	IRI	82.5	40.9	7	117.0	19.4	7	34.5	68.1	0.9
Liang et al., (2015)^58^*	miR-26a	IRI	108.8	11.7	6	58.8	5.2	6	− 50.0	− 39.8	− 60.2
Lan et al., (2012)^67^*	miR-494	IRI	65.9	32	7	135.5	82.5	7	69.6	135.2	4.0
	Total		118.5	29.8	74	101.3	45.0	75	− 17.2	− 5.0	− 29.5
Wei et al., (2020)^4^*	miR-21	Sepsis	48.4	19.9	4	77.9	17.4	4	29.5	55.4	3.6
Funahashi et al., (2019)^9^*	miR-146a	Sepsis	47.2	90.3	7	13.9	7.3	7	− 33.3	33.8	− 100.4
Li et al., (2018b)^25^*	miR-124	Sepsis	152.5	19.4	20	45.2	10.4	20	− 107.3	− 97.7	− 116.9
	Total		82.7	54.5	31	45.7	12.4	31	− 37.0	− 17.3	− 56.7
**Antagomir**											
Geng et al., (2020)^2^*	miR-21	IRI + IPC	67.6	23.3	6	145.72	36.6	6	78.1	112.8	43.4
Zhao et al., (2020)^7^*	miR-27a-3p	IRI	116.1	38.6	3	91.9	19.2	3	− 24.2	24.6	− 72.9
Li et al., (2019)^10^*	miR-23a	IRI	111.3	8.2	5	99.3	3.78	5	− 12.0	− 4.1	− 19.9
Liu et al., (2019)^11^*	miR-377	IRI	209.2	44	8	156.5	60	8	− 52.7	− 1.1	− 104.3
Song et al., (2018a)^28^*	miR-21	IRI	223.3	50.7	6	274.4	54.1	6	51.1	110.4	− 8.2
Wei et al., (2018)^30^*	miR-668	IRI	178.2	37.8	6	239.9	28	7	61.7	97.5	25.9
Huang et al., (2018)^38^*	miR-146	IRI	59.0	8.2	6	74.4	4	6	15.4	22.7	8.1
Jiao et al., (2017)^40^*	miR-21	IRI + IPC	45.1	6.6	5	113.24	28.5	5	68.1	93.8	42.5
Xu et al., (2017a)^45^*	miR-21	IRI + IPC	41	17.2	6	111.1	22.5	6	70.1	92.8	47.4
Xu et al., (2017b)^46^*	miR-21	IRI + CoCl_2_ PC	71.9	33.3	6	129.8	111.8	6	57.9	151.2	− 35.4
Chen et al., (2016a)^49^*	miR-16	IRI	93.1	31.1	7	75.5	21.8	7	− 17.6	10.5	− 45.7
Dai et al., (2016)^51^*	miR-146a	IRI + LPS PC	54.2	10.3	6	158.6	27.8	6	104.4	128.1	80.7
Wei et al., (2016)^54^*	miR-489	IRI	74.5	30.6	3 to 7	82	33.1	3 to 7	7.5	40.9	− 25.9
Bhatt et al., (2015)^55^*	miR-687	IRI	227.9	8	4	104.3	35.6	4	− 123.6	− 87.8	− 159.4
Lorenzen et al., (2014)^63^*	miR-24	IRI	225.7	12.7	20	121.7	21.9	20	− 104.0	− 92.9	− 115.1
Jia et al., (2013)^65^*	miR-21	IRI + Xe PC	63.3	12.1	5	118.6	25.9	5	55.3	80.4	30.2
Lan et al., (2012)^67^*	miR-494	IRI	86.9	19.2	5	55.4	21.5	5	− 31.5	− 6.2	− 56.8
Xu et al., (2012)^69^*	mir-21	IRI	35.4	1.9	6	33.4	4.9	5	− 2.0	2.2	− 6.2
Xu et al., (2012)^69^*	miR-21	IRI + IPC	18.6	2.8	6	26.9	3.8	5	8.3	12.2	4.4
	Total		105.4	25.5	123	116.5	52.4	122	11.1	21.4	0.8
Wei et al., (2020)^4^*	miR-21	Sepsis	50.1	6.5	4	31.1	18.5	4	− 19.0	0.2	− 38.2
Wang et al., (2017b)^43^*	miR-107	Sepsis	81.4	5.7	5	48.7	6	5	− 32.7	− 25.4	− 40.0
Jia et al., (2017)^39^*	miR-21	Sepsis + IPC	48.6	15.2	6 to 8	61.9	14	6 to 8	13.3	27.6	− 1.0
Jia et al., (2015)^57^*	miR-21	Sepsis + Xe PC	39.4	9.7	6	53.8	17.8	6	14.4	30.6	− 1.8
	Total		54.9	10.0	23	48.9	14.9	23	− 6.0	1.3	− 13.3
**Gene deletion**											
Wang et al., (2020)^3^*	miR-218-5p	IRI	22.7	5.7	8	208.8	86.2	9	186.0	246.1	126.0
Yan et al., (2020a)^5^*	miR-214	IRI	170.7	61.4	> 6	96.38	35.37	> 6	− 74.3	− 17.6	− 131.0
Ranganathan et al., (2015)^60^*	miR-150	IRI	142.9	12	6	83.8	13.9	6	− 59.1	− 44.4	− 73.8
Wei et al., (2010)^70^*	Multiple	IRI	197	72	4	69.1	28	4	− 127.9	− 52.2	− 203.6
	Total		133.3	47.8	24	114.5	49.1	25	− 18.8	8.3	− 46.0
Pan et al., (2019)^13^*	miR-21	Sepsis + IPC	44.9	10.3	4	141.4	9.7	4	96.5	110.4	82.6

*AKI* acute kidney injury, *CI* confidence interval, *CoCl*_*2*_* PC* cobalt chloride preconditioning, *IPC* ischemic preconditioning, *IRI* ischemia reperfusion injury, *LPS PC* lipopolysaccharide preconditioning, *miRNA* micro-RNA, *n* sample size, *NS* not specified, *SCr* serum creatinine, *SD* standard deviation, *Xe PC* xenon preconditioning.

*Study references are found in the Supplementary materials; + 5 studies in mice did not report creatinine and were excluded.

To determine the impact of the injury model (IRI and sepsis) on the overall effect for each intervention, we calculated the degree of overlap between the 95% CIs estimated from the group average for the individual mean differences. The average of the individual mean differences between IRI and sepsis models that used miRNA mimics as intervention was associated with a 50% overlap in the 95% CIs, compared to only 7% with antagomirs. However, the trends for these differences were non-significant for both models (*p* > 0.05).

### Biologic processes

**Figure 3:**
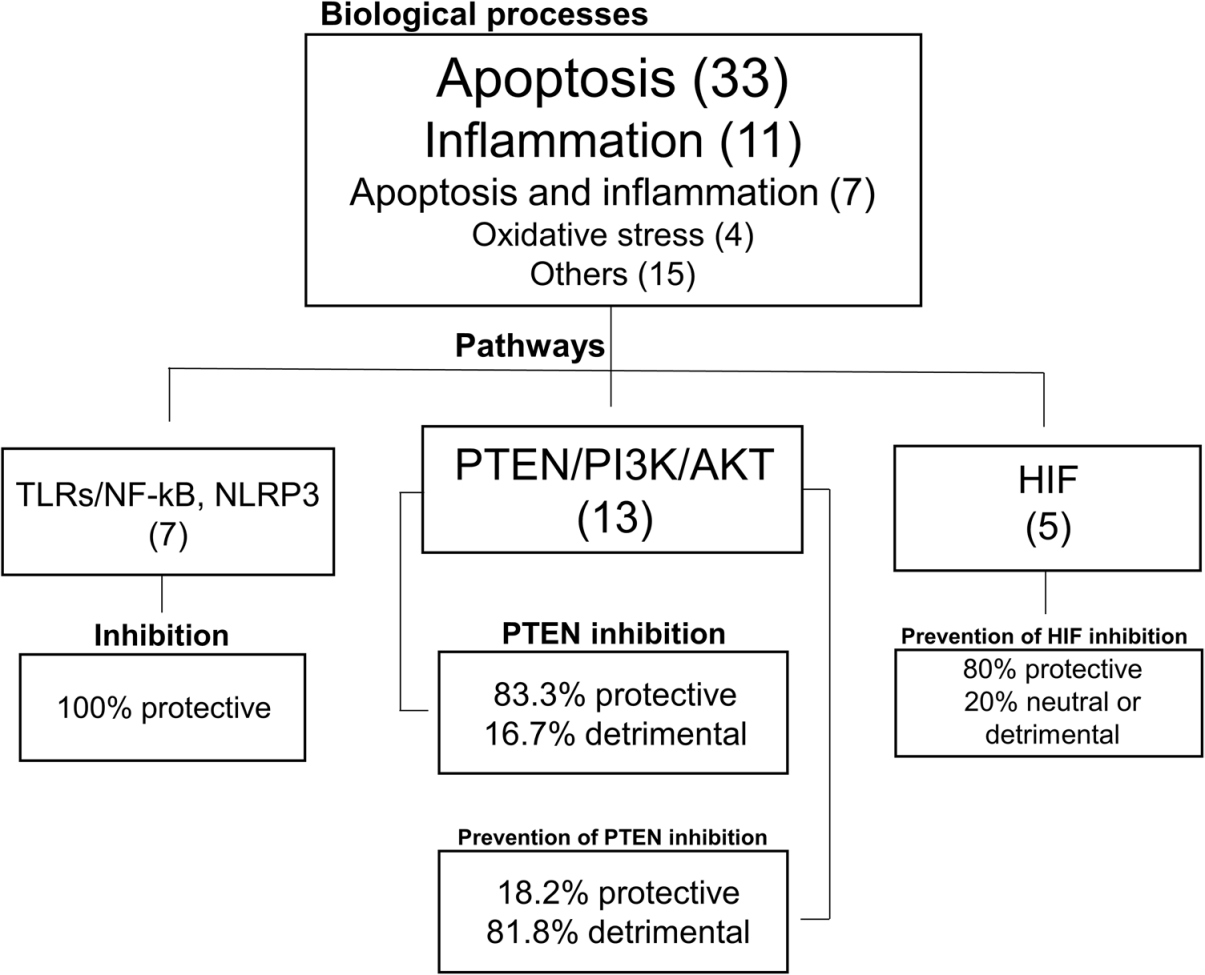
Schematic representation of main biologic processes and signaling pathways in included studies. Apoptosis was the most common biologic process targeted by the interventions and PTEN/PI3K/AKT was the main pathway. AKT: Protein kinase B, HIF: Hypoxia-inducible factor, NF-κB: nuclear factor kappa B, NLRP3: Nod-like receptor family pyrin domain containing 3, PI3K: Phosphoinositide 3-kinase, PTEN: phosphatase and tensin homolog, and TLR: Toll-like receptor.

Seventeen separate biological processes were reported within the studies as potential targets of the miRNA intervention in AKI. The majority involved at least one of three processes (Fig. 3): apoptosis (47.1%), inflammation (15.7%), or oxidative stress (5.7%). Apoptosis and inflammation overlapped in 10.0% of studies. *Phosphatase and tensin homolog* (PTEN)/AKT/mammalian target of rapamycin (mTOR) was the most frequent signaling pathway implicated (18.6%), with linkage to apoptosis. Of the 13 studies implicating PTEN (and apoptosis) in mediating effects of the miRNA intervention, 7 involved miRNA-21, and 5 were from one group of investigators (38.0%). Toll-like receptor (TLR)/NF-kB/NLPR3 signaling was studied in 10.0%, while 7.1% of studies involved the hypoxia-inducible factor (HIF)/vascular endothelial growth factor (VEGF) pathway. Interventions inhibiting PTEN were protective in 83.3% of studies, whereas treatments preventing PTEN inhibition or upregulating it were detrimental in 81.8%***.*** miRNA interventions against TLRs/NF-kB attenuated inflammatory responses, protecting against impaired kidney function and morphological injury in all studies examining this pathway. Interventions that promoted stimulation of the HIF pathway were protective in 80.0% of studies.

### Adverse effects and survival data

No studies reported on adverse effects or off-target consequences of the miRNA intervention on other organs, and only 9 studies (12.9%) provided rodent survival data.

### Meta-analyses of miRNA-21

**Figure 4:**
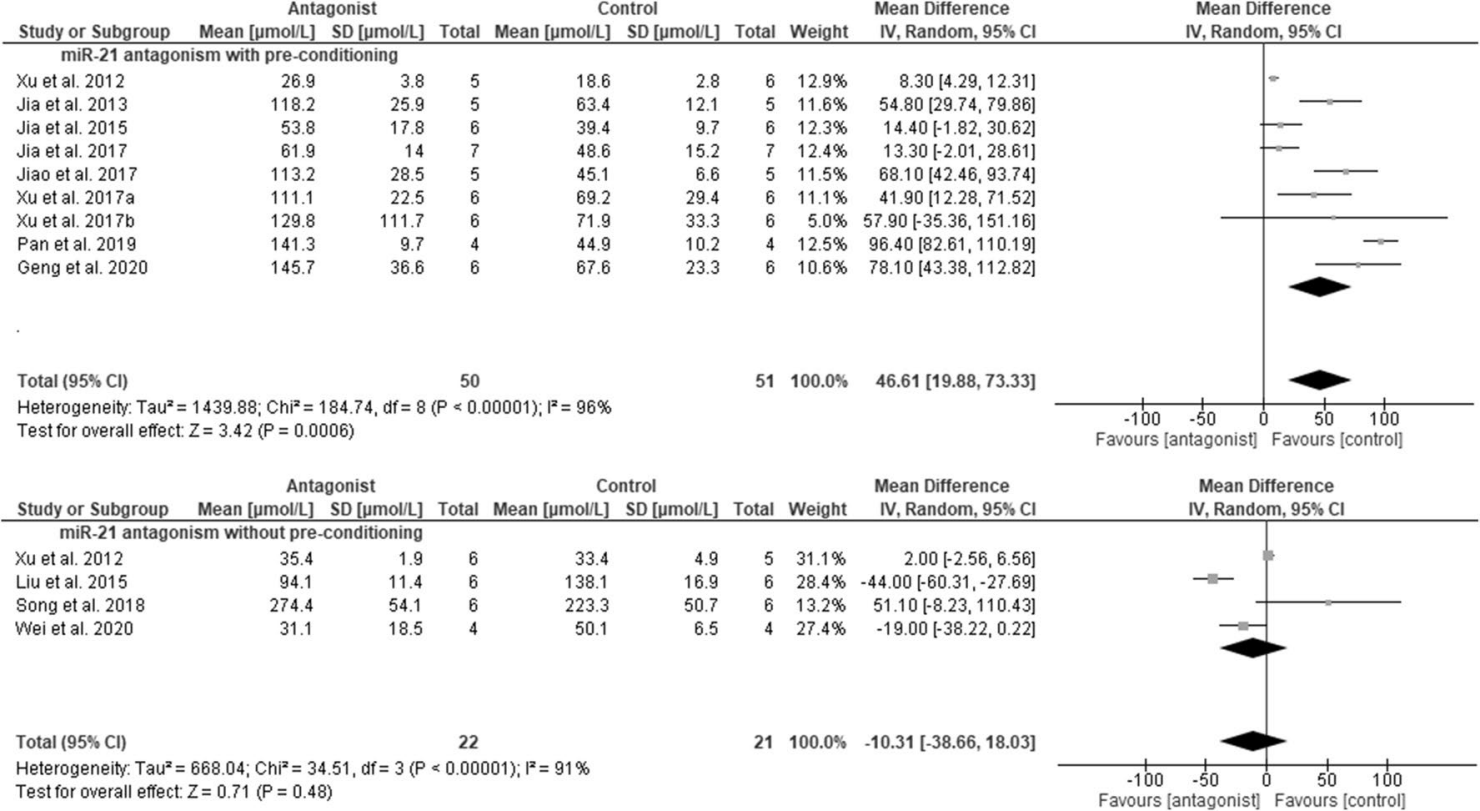
Meta-analyses of miRNA-21 antagonism on serum creatinine in AKI models (in the presence or absence of pre-conditioning). SD, standard deviation; CI, confidence interval.

**Figure 5:**
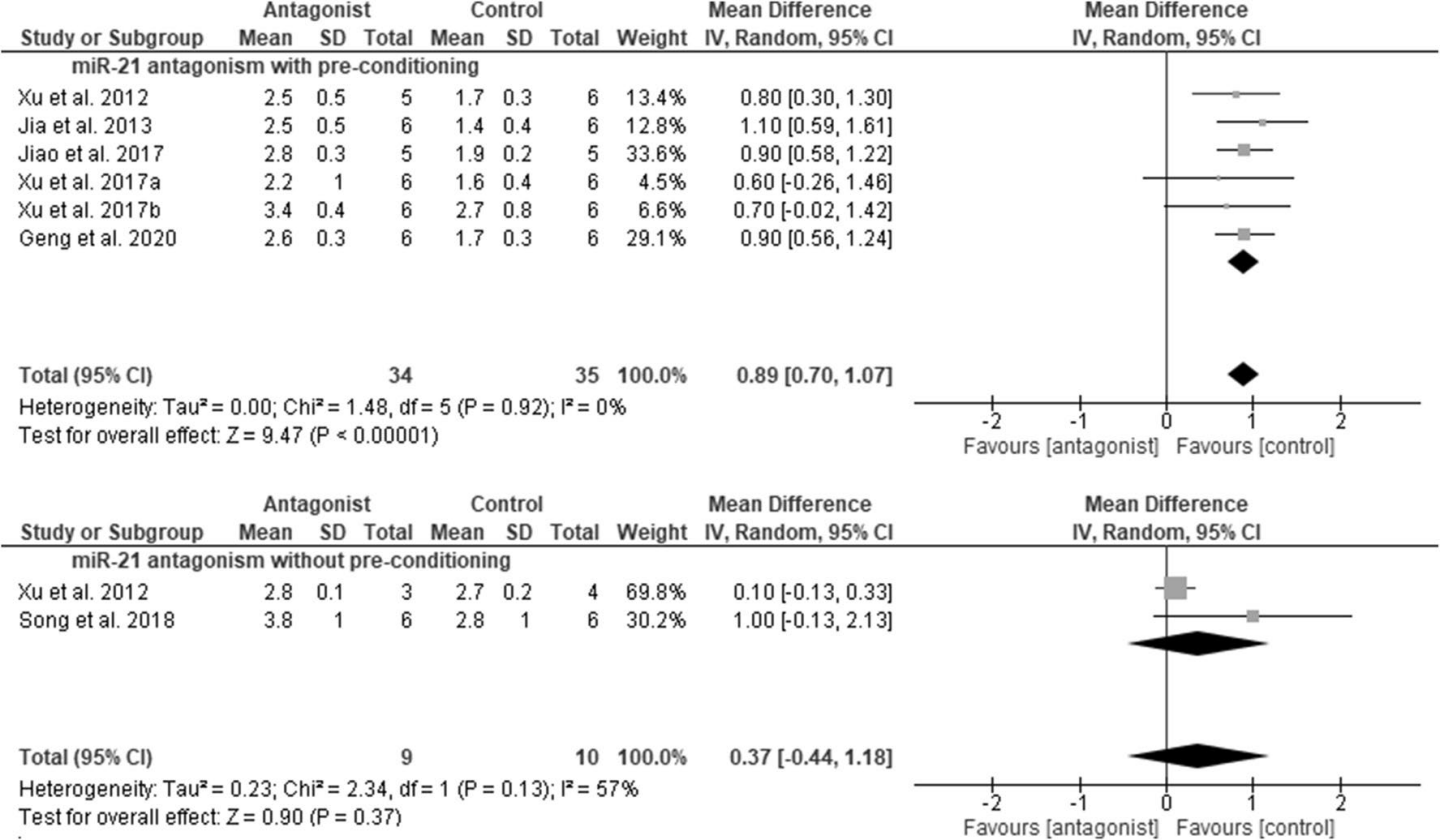
Meta-analyses of miRNA-21 antagonism on injury scores in AKI models (in the presence or absence of pre-conditioning). SD, standard deviation; CI, confidence interval.

Fourteen studies focused on the effects of miRNA-21 in AKI. Of these, 12 studies involved administration of miRNA-21 antagomirs (n = 2), locked nucleic acid (LNA) (n = 9) against miRNA-21, or gene deletion (n = 1), and were included in a meta-analysis. Twelve studies reported serum Cr levels, and 7 included histologic kidney injury scores. Pooling these studies was done independently of differences in study design, AKI model, or species characteristics. Nine of the 12 studies involved ischemic, xenon gas or cobalt chloride pre-conditioning along with miRNA-21 antagonism in the AKI model. All 9 studies involved investigators from one research group. With pre-conditioning, miRNA-21 antagonism significantly increased serum Cr levels (mean difference in Cr: 46.6 µmol/L; 95%CI 19.9, 73.3 µM; *p* = 0.0006), although study heterogeneity was high (*p* < 0.00001) (Fig. 4). With pre-conditioning in AKI, kidney injury scores were significantly increased by miRNA-21 antagonism (mean difference in score: 0.89; 95%CI 0.70, 1.07; *p* < 0.00001), and heterogeneity was low (*p* = 0.92) (Fig. 5).

Among mouse studies involving miRNA antagonism with pre-conditioning, 6 included IRI models, and 3 involved sepsis. For IRI studies, the mean difference in serum Cr associated with the miRNA-21 intervention was 56.3 µM (95% CI 37.9, 74.7), while for sepsis models, the mean difference in serum Cr was 41.4 µM (95% CI 32.8, 50.0), with 70% overlap in 95% CIs. By unpaired t-test, there was no significant difference in effect size according to model (*p* > 0.05).

Four studies involved administration of antagomir to miRNA-21 in the AKI model, in the absence of pre-conditioning. Three studies involved IRI models and 1 involved sepsis. Due to limited study numbers, calculation of comparative effect of models on changes in serum Cr was not performed.

Meta-analysis of studies in the absence of pre-conditioning revealed no significant effect of miRNA-21 antagonism on serum Cr (*p* = 0.48), although heterogeneity was high (*p* < 0.00001). Kidney injury scores were reported in only 2 of these studies, were not affected overall (*p* = 0.37), and heterogeneity was insignificant (*p* = 0.13).

Interestingly, 9 of the 12 studies included in the meta-analysis of miRNA-21 antagonism reported apoptosis as the predominant biologic process, with 4 of 12 studies implicating PTEN as the pathway target.

### Effect of miRNA-146a

Four studies focused on effects of miRNA-146a in AKI, and 3 of these involved delivery of miRNA-146a antagomirs. Dai et al. showed that lipopolysaccharide (LPS) pretreatment protected mice from kidney IRI, and this effect was abolished by knockdown of miRNA-146a by administration of locked nucleic acid anti-miRNA^19^. Huang et al. used both mimic and antagomir to show protective effects of miRNA-146a in mice with kidney IRI^20^, and Amrouche et al. demonstrated significant increases in kidney tubular injury scores in mice with miRNA-146a gene deletion and unilateral kidney IRI^21^. Finally, in a cecal ligation and puncture model of sepsis-induced AKI in mice, Funahashi et al. reported that administration of miRNA-146a expression plasmid decreased serum Cr and prevented multi-organ injury by targeting splenic macrophages^22^. The limited number of studies and model heterogeneity precluded conduct of a meta-analysis, although the data suggest an overall protective effect of miRNA-146a in experimental AKI.

### Effect of miRNA-155

Three studies (2 in mice, 1 in rat) examined the effects of miRNA-155 in AKI. Two studies administered antagomir and showed a protective effect in IRI- or LPS-induced AKI^23,24^. By contrast, a third study reported that gene deletion of miRNA-155 further aggravated cisplatin-induced nephrotoxic injury^25^. Insufficient outcome data and high statistical heterogeneity prevented meta-analysis.

### Quality assessment

**Figure 6:**
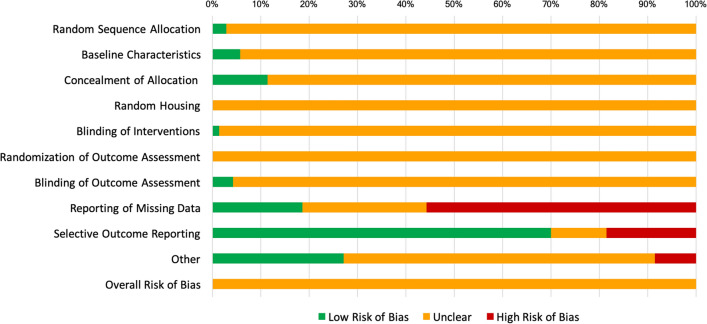
Risk of bias for the 70 included studies, using the SYRCLE ROB tool. Low risk of bias is represented in green, unclear risk in orange and high risk of bias in red.

Quality of evidence for studies involving miRNA-21 across all interventional categories was deemed very low (Supplementary Table S2). All studies were assessed overall as having unclear risk of bias (Fig. 6), due to the lack of either explicit randomization methods (97.1%), explicit blinding to the interventions (98.6%), or blinding of the outcome assessors (95.7%). In some studies, quality was unclear since there was no report of miRNA dose (12.9%), or age or weight of the animals (18.6%).

### Publication bias

In considering the unclear risk of bias in most studies, the presence of high statistical heterogeneity (> 80%), and the limited number of studies (< 20) included in the quantitative analyses, we concluded that a report on publication bias could be misleading. Thus, scatter plot asymmetry and statistical tests on reporting bias were not conducted.

## Discussion

This systematic review and meta-analysis identified 70 pre-clinical studies addressing 42 distinct miRNA variants as therapeutic targets in AKI. Several major findings deserve emphasis: first, despite the significant heterogeneity in design features, all studies reported significant effects of the miRNA intervention (protective or detrimental) on kidney functional or structural outcomes, with the majority reporting an effect on serum Cr levels. While collectively these studies support a prominent role for miRNAs in AKI pathogenesis and therapeutic application, under-reporting of negative studies must also be considered as a potential confounding factor. Second, a meta-analysis of studies with miRNA-21 antagonism indicates that miRNA-21 significantly protects against injury in pre-clinical AKI models accompanied by ischemic, xenon or cobalt chloride pre-conditioning, without significant difference in effect size according to intervention, or in IRI compared to sepsis models. Third, although the number of studies is limited (n = 4), miRNA-146a appears to exert protective effects in mouse models of sepsis or IRI AKI. Fourth, beneficial effects of miRNA interventions were most frequently associated with prevention of apoptosis as a protective mechanism, and with inhibition of PTEN as a dominant signaling pathway. Finally, the overall quality of studies was found to be low, with limitations in study design and data reporting that reduced the overall strength of evidence.

miRNA sequences are highly conserved across species, and tissue distribution is similar between rodents and humans^26^. Numerous miRNAs are endogenously expressed in mammalian kidneys, and expression patterns are altered in disease states^9,26^. The biogenesis of miRNAs is complex, involving canonical and non-canonical pathways starting with generation of pri-miRNA transcripts or small hairpin RNAs within the cell nucleus, respectively, followed by export to the cytoplasm where further processing occurs to create a miRNA-induced silencing complex (miRISC)^27^. The miRNA then interacts with the 3′UTR of target mRNAs and inhibits translation or induces mRNA degradation^27^. In the present study, we uncovered a substantial number of miRNA variants (n = 42) reported to have significant effects on kidney function and indices of structural injury in experimental AKI. The diversity of miRNA variants strongly suggests converging effects on molecular signaling targets with downstream impacts on common biological processes involved in kidney injury and repair. Indeed, the PTEN/AKT/mTOR signaling pathway was the most frequent target, with inhibition of PTEN protective in a majority of the studies, and with subsequent benefits on apoptosis and inflammation. However, several studies implicating PTEN were conducted by a single research group, indicating a potential source of bias. The mechanisms whereby miRNAs identified in our review converge on common signaling events in AKI could involve binding to multiple distinct gene targets within these pathways. An alternate hypothesis is that variable affinity of miRNA-mRNA interactions could play a role, dependent on miRNA dose or delivery systems in vivo. Furthermore, interactions of miRNAs with 5′UTRs, coding sequences, or gene promoters (with activation of gene expression), have also been described^26^, and might account for the potent regulatory effects in AKI.

miRNA-21 is expressed in the kidney, heart and lungs in mammals, and could serve as a clinical biomarker, as it has been detected in human plasma and urine^28,29^. Kidney and urine levels of miRNA-21 are elevated in rats with AKI or hypertensive injury, also supporting a biomarker role^15,30,31^. Our review identified 14 studies directed at the functional effects of miRNA-21 in pre-clinical AKI, representing the miRNA most frequently investigated. In the meta-analyses of studies with preconditioning, both serum Cr and injury scores were significantly increased in studies involving miR-21 antagonism as the intervention, supporting the hypothesis that endogenous miRNA-21 prevents kidney injury. Indeed, preconditioning appears to upregulate kidney miRNA-21 expression, which may be linked to its protective effect in AKI. Inhibition of miRNA-21 worsened apoptosis in most studies, and a third of studies identified PTEN as a key target. The data suggests that miRNA-21 may block apoptosis by downregulating several other targets, including PDCD4, NF-kB, tumor necrosis factor (TNF), interleukin (IL)-6, and via activation of the pro-survival AKT pathway or upregulation of *B-cell lymphoma* (Bcl)-2^16,32^. Interestingly, while 2 of 4 studies involving miRNA-21 antagonism without pre-conditioning showed neutral or adverse effect on kidney function, 2 studies reported improvements in serum Cr^33,34^. Potential causes for these disparate results are unclear, although the detrimental or neutral effects of miRNA antagonism in AKI were limited to multiple reports from a single research group. We acknowledge that model characteristics, dose, and timing of interventions may also have contributed to these differences in outcomes. Finally, it is important to note that few studies examined the direct effects of miRNA-21 mimic or overexpression in AKI, and meta-analysis was restricted to studies with miRNA-21 antagonism. Further research should examine the potential protective effects of direct administration of miRNA-21 in these experimental AKI models.

miRNA-146a has been investigated as a biomarker in human subjects with AKI and as a therapeutic agent in pre-clinical models^12^. In mice with gene deletion of miRNA-146a, kidney injury and inflammation increased after IRI, with the proposed pathway targeting IL-1 receptor-associated kinase 1 (IRAK1)^21^. Three studies also demonstrated a role for miRNA-146a in targeting IRAK1, and protecting against detrimental inflammatory responses^19,21,22^. Despite these findings, due to the limited number of studies evaluating miRNA-146 and the heterogeneity between studies, a meta-analysis was not performed here. Nonetheless, data from the 4 studies involving miRNA-146a support the protective properties of this miRNA in experimental AKI, the role of IRAK targeting, and the potential for translation to application in human AKI.

This study has several strengths, including adoption of a comprehensive search strategy, rigorous data abstraction methodology, detailed performance of quality assessments, and inclusion of two independent reviewers at each step. Limitations include the overall low quality of evidence, and the attribution of unclear risk of bias to most studies, due in part to limited detail within manuscripts on blinding or randomization procedures. Several studies did not provide sufficient information on study population characteristics or interventions, and attempts to contact study authors were not successful. Importantly, studies were limited in population diversity, since they were exclusively performed in rodents, with a majority restricted to males. IRI was the most common AKI model, which may not closely mimic the disease condition in humans. Finally, of the 70 studies, none reported on adverse effects of the miRNA intervention or impact on function of organs other than kidney, which underscores a deficiency in pre-clinical experimental design. Due to these limitations, the significance of the pre-clinical data for application to human disease remains unclear. Few studies in our review described rodent populations with comorbidities, while human AKI often presents clinically with other conditions such as hypertension, pre-existing kidney disease, diabetes mellitus, or heart failure^35^. Accordingly, our study and other reports^36^ highlight the critical need for attention to risk of bias, enhanced diversity of model characteristics, and examination of adverse effects in pre-clinical AKI.

While challenges remain in translating findings from pre-clinical studies for application to human AKI, it is noteworthy that human clinical trials involving miRNA therapeutics have been completed, or are currently in progress. In patients with advanced solid tumors, including renal cell carcinoma, a Phase 1 trial of i.v. administration of liposomal miRNA-34a mimic was found to target tumors and modulate gene expression in white blood cells, although serious immune-mediated adverse events led to early trial closure^37^. A Phase I clinical trial is underway in patients with Alport nephropathy, involving administration of compound RG-012, which silences miRNA-21 and may lead to alterations in glomerular basement membrane protein structure (ClinicalTrials.gov, NCT03373786). The anti-miRNA-17 oligonucleotide RGLS4236 has recently been shown to preferentially distribute to kidneys in mice after with polycystic kidney disease (PKD) after s.c. injection, and it inhibits cyst growth in several PKD mouse models^38^. Since this miRNA-17 inhibitor also attenuates growth of human cysts in vitro, it holds considerable promise as a treatment for autosomal dominant PKD. Accordingly, further well-designed pre-clinical studies involving miRNA therapeutics in AKI may advance the design and conduct of Phase I trials in humans with AKI.

## Materials and methods

The systematic review was conducted in accordance with the Cochrane Collaboration Methods, Systematic Reviews Standards^39,40^ and PRISMA guidelines^40^. The study protocol has been published^18^ and registered in PROSPERO (www.crd.york.ac.uk/prospero) (CRD42019128854).

### Search strategy

A comprehensive search strategy used MEDLINE, EMBASE, OVID, PUBMED and Web of Science search engines for studies published from 1946 to April 30, 2020. MesH terms used were related to miRNAs, exosomes, extracellular vesicles and AKI^18^ (Supplementary File 1).

### Inclusion and exclusion criteria

Included studies had the following characteristics: (a) Population: preclinical model of AKI and in vivo mammalian (non-human) model; (b) Intervention: Direct administration of miRNA mimics, antagomirs, locked nucleic acids or gene deletion. In vivo studies that did not involve direct administration of miRNA mimic/antagomir, but implicated miRNAs mechanistically in AKI were also included as “indirect” interventions; (c) Comparator: any type of comparator, and (d) Outcome measure: clearly identified kidney function and/or structural marker(s) of AKI. Secondary outcomes consisted of adverse effects of miRNA and mortality. Studies were excluded if they did not involve use of a mammalian, non-human in vivo experimental model of AKI (e.g. in vitro cell culture alone), if there was no intervention involving administration of miRNA or derivatives (or no mechanistic information related to miRNA), or if there were no outcome measures of kidney function or structural injury. Studies were also excluded if they were not considered as original research (e.g. review articles, commentaries), or if the language was not English, French, Spanish or Italian.

### Study screening

Titles and abstracts were initially screened for relevance and manuscripts were then selected for full text review by 2 independent reviewers. In cases of disagreements between the 2 reviewers, resolution occurred by consensus after discussion, or by involvement of a third independent reviewer.

### Data extraction

Data was abstracted by 2 independent investigators using a piloted and standardized form in Research Electronic Data Capture (REDCap)^41,42^. Extracted data included: (1) study characteristics, design, and methods: title, authors, journal/year, language, country, randomization, allocation, concealment and blinding methods (where applicable); (2) population characteristics: animal species and strain, sex, weight, age, total number, AKI model, comorbidities if present; (3) interventions: miRNA species and type of intervention, dose, frequency, timing, and route of administration; (4) functional/structural outcomes: serum Cr, BUN or urea, GFR, Cr clearance or other reported functional outcomes, and kidney injury scores, inflammatory cell infiltration, apoptosis measures or other histological assays, as well as sex-specific data, mortality, and adverse effects associated with the intervention. If study data were incomplete, attempts were made to contact the corresponding author. ImageJ software^43^ was used to extract study data.

### Quality assessment

Study quality was evaluated by 2 independent reviewers using the SYRCLE Risk of Bias tool for animal studies^44^. To assess the certainty in the evidence and strength of recommendations for the meta-analysis findings in this review, 2 reviewers evaluated quality of evidence according to 5 domains of GRADE recommendation^45^ (Supplementary Table S3).

### Data analysis

Results were analyzed in Review Manager 5.3^46^. For each study, both the mean difference (MD) and standardized mean difference (SMD) were calculated using the group means and their standard deviations. If different measures of central tendency and distribution were available, means and standard deviations were estimated according to described algorithms^47,48^. Studies in which clinical heterogeneity was deemed to be acceptable were selected for quantitative analysis (i.e. meta-analysis). In the meta-analysis, variation between studies was evaluated by Forest plots and I^2^ statistics. To obtain a pooled effect estimate from all studies, a weighted average of the intervention effects from individual studies was calculated by the inverse-variance method (*p* < 0.05) and data was conservatively modeled according to the random effects model^49^. We assessed the impact of potential sources of bias on the pooled effect estimates by using sensitivity and sub-group analyses^50–52^. Since there was variability in the time of measurement post-AKI induction, we selected the maximum effect estimate within 48 h of AKI induction as outcome measure. Studies that did not provide adequate data for quantitative analysis were assessed descriptively.

## Supplementary information

Supplementary Information.
