# Supplementary material for: Therapeutic effects of micro-RNAs in preclinical studies of acute kidney injury: a systematic review and meta-analysis
Source: Sci Rep. 2021 Apr 27;11:9100. doi: 10.1038/s41598-021-88746-y (PMC8079678; doi:10.1038/s41598-021-88746-y)
Supplement: Supplementary file 1 — Supplementary Information. [file 41598_2021_88746_MOESM1_ESM.pdf]

Therapeutic Effects of Micro-RNAs in Preclinical Studies of Acute Kidney Injury: A Systematic Review and Meta-Analysis

Sarah Zankar, Mayra Trentin-Sonoda, Jose L. Viñas, Rosendo A. Rodriguez, Adrian Bailey, David Allan and Kevin D. Burns

Supplementary information

Supplementary Table S1. Characteristics of studies included in the systematic review

Study	AKI model	Species	Sex	miRNA	Type of Intervention	Route of administration	SCr, BUN or Injury Scores	Pathway Category	Intervention Outcome
Du et al., 2020 [1]	IRI	Rats	Male	miR-21	MVs	I.V.	No	Apoptosis	Detrimental
Geng et al., 2020 [2]	IRI + IPC	Mice	Male	miR-21	Gene deletion, antagomiR	Gene deletion, I.P.	Yes	Apoptosis	MiR-21 tg +/0: Protective; AntagomiR: Detrimental
Wang et al., 2020 [3]	IRI	Mice	NS	miR-218-5p	Gene deletion, mimic	Gene deletion, I.V.	Yes	Angiogenesis	Detrimental
Wei et al., 2020 [4]	Sepsis	Mice	Male	miR-21	Mimic, antagomiR	I.V.	Yes	Apoptosis	Mimic: Detrimental; AntagomiR: Protective

Study	AKI model	Species	Sex	miRNA	Type of Intervention	Route of administration	SCr, BUN or Injury Scores	Pathway Category	Intervention Outcome
Yan et al., 2020a [5]	IRI	Mice	Male	miR-214	Gene deletion	Gene deletion	Yes	Apoptosis	Protective
Yan et al., 2020b [6]	Acute Pancreatitis	Rats	Male	miR-214-3p	Mimic, antagomiR	I.V.	Yes	Inflammation	Mimic: Detrimental; AntagomiR: Protective
Zhao et al., 2020 [7]	IRI	Mice	Male	miR-27a-3p	Mimic, antagomiR	I.V.	Yes	Oxidative stress	Mimic: Detrimental; AntagomiR: Protective
Chen et al., 2019 [8]	IRI	Mice	NS	miR-424	Mimic	I.V.	Yes	Apoptosis	Protective
Funahashi et al., 2019 [9]	Sepsis	Mice	Male	miR-146a	Mimic	I.V.	Yes	Inflammation	Protective
Li et al., 2019 [10]	IRI	Mice	Male	miR-23a	Exosomes, antagomiR	I.V.	Yes	Inflammation	Exosomes: Detrimental; AntagomiR: Protective
Liu et al., 2019 [11]	IRI	Mice	Male	miR-377	Mimic, antagomiR	I.V.	Yes	Inflammation	Mimic: Detrimental; AntagomiR: Protective
Ma et al., 2019 [12]	Sepsis	Mice	Male	miR-590-3p	Mimic	I.V	Yes	Apoptosis, inflammation	Protective
Pan et al., 2019 [13]	Sepsis + IPC	Mice	Male	miR-21	Gene deletion	I.V	Yes	Apoptosis, inflammation	Gene deletion: Detrimental

Study	AKI model	Species	Sex	miRNA	Type of Intervention	Route of administration	SCr, BUN or Injury Scores	Pathway Category	Intervention Outcome
Qin et al., 2019 [14]	Sepsis	Rats	Female	miR-191-5p	Mimic	I.V	Yes	Apoptosis, inflammation	Protective
Tapparo et al., 2019 [15]	Rhabdomy-olysis	Mice	Male	miR-10a, miR-127 and miR-486	EVs	I.V.	Yes	Unclear	Protective
Wu et al., 2019 [16]	Allograft renal transplanta-tion	Rats	Male	miR-191	AntagomiR	Intra-rectal	No	Apoptosis	Protective
Yue et al., 2019 [17]	IRI	Rats	Male	miR-187	Mimic	I.V.	Yes	Apoptosis	Protective
Zhang et al., 2019a [18]	Nephrotoxic + IPC	Mice	Male	miR-144	AntagomiR	I.V.	No	Apoptosis	Detrimental
Zhang et al., 2019b [19]	IRI	Rats	Male	miR-155	AntagomiR	NS	Yes	Apoptosis	Protective
Zhang et al., 2019c[20]	IRI	Rats	NS	miR-30c-5p	Mimic	I.V.	Yes	Inflammation	Protective
Zhu et al., 2019 [21]	IRI	Mice	Male	miR-204-5p	Mimic	I.V.	Yes	Apoptosis, necrosis	Protective
Chen et al., 2018 [22]	Sepsis	Mice	NS	miR-155	AntagomiR	I.V.	No	Inflammation	Protective
Guo et al., 2018 [23]	Nephrotoxic	Mice	Male	miR-709	AntagomiR	I.P.	Yes	Mitochondrial function, apoptosis	Protective
Li et al., 2018a [24]	IRI	Rats	Male	miR-182	AntagomiR	I.V.	Yes	Apoptosis	Protective

Study	AKI model	Species	Sex	miRNA	Type of Intervention	Route of administration	SCr, BUN or Injury Scores	Pathway Category	Intervention Outcome
Li et al., 2018b [25]	Sepsis	Mice	Male	miR-124	Mimic	I.V.	Yes	Inflammation	Protective
Liu et al., 2018 [26]	Nephrotoxic	Mice	Male	miR-181a	AntagomiR	I.V.	Yes	Apoptosis	Protective
Mathia et al., 2018 [27]	Rhabdo-myolysis	Mice	Male	miR-22	AntagomiR	S.C.	Yes	Oxidative stress	Detrimental
Song et al., 2018a [28]	IRI	Mice	Male	miR-21	AntagomiR	I.V.	Yes	Apoptosis, inflammation	Detrimental
Song et al., 2018 [29]	IRI	Mice	Male	multiple including miR-17-92 cluster	Gene deletion, mimic	I.V.	Yes	Apoptosis, cell proliferation	Protective
Wei et al., 2018 [30]	IRI	Mice	Male	miR-668	Mimic, antagomiR	I.V.	Yes	Mitochondrial dynamics	Mimic: Protective; AntagomiR: Detrimental
Yu et al., 2018 [31]	DHCA	Rats	Male	miR-106b-5p	Mimic	Intra-Renal	No	Apoptosis	Protective
Zhu et al., 2018 [32]	IRI	Rats	Male	miR-214	Mimic	I.P.	Yes	Apoptosis	Protective
Amrouche et al., 2017 [33]	IRI	Mice	Male	miR-146a	Gene deletion	Gene deletion	Yes	Inflammation	Detrimental
Colbert et al., 2017 [34]	Sepsis (CLP, LPS, FS and MRSA)	Mice	Male	miR-223	Gene deletion	Gene deletion	Yes	NS	CLP: Protective; LPS: Detrimental; FS: Neutral; MRSA: Neutral
de Almeida et al., 2016 [35]	Nephrotoxic	Mice	Male	miR-377, miR-141	MSCs, MVs	I.P.	Yes	Multiple	Protective
Fu et al., 2017 [36]	Sepsis	Rats	Female	miR-21	Mimic	I.V.	Yes	Apoptosis	Protective
Hao et al., 2017 [37]	IRI	Mice	Male	miR-17-5p	Mimic	I.V.	Yes	Apoptosis	Protective

Study	AKI model	Species	Sex	miRNA	Type of Intervention	Route of administration	SCr, BUN or Injury Scores	Pathway Category	Intervention Outcome
Huang et al., 2018 [38]	IRI	Mice	Male	miR-146	Mimic, antagomiR	NS	Yes	Apoptosis	Mimic: Protective; AntagomiR: Detrimental
Jia et al., 2017 [39]	Sepsis + IPC	Mice	NS	miR-21	AntagomiR	I.P.	Yes	Apoptosis, inflammation	Detrimental
Jiao et al., 2017 [40]	IRI + IPC	Mice	Male	miR-21	AntagomiR	I.V.	Yes	Oxidative stress	Detrimental
Ranghino et al., 2017 [41]	IRI	Mice	Male	multiple	EVs	I.V.	Yes	Multiple	Protective
Wang et al., 2017a [42]	Nephrotoxic	Mice	Male	miR-301a-5p	AntagomiR	I.V.	Yes	Apoptosis	Protective
Wang et al., 2017b [43]	Sepsis	Mice	Male	miR-107	AntagomiR	I.V.	Yes	Apoptosis	Protective
Wilflingseder et al., 2017 [44]	IRI	Rats	Male	miR-182-5p	AntagomiR	I.V.	Yes	Cell cycle, inflammation	Protective
Xu et al., 2017a [45]	IRI + IPC	Mice	Male	miR-21	AntagomiR	I.V.	Yes	Apoptosis	Detrimental
Xu et al., 2017b [46]	IRI + $CoCl_2$ PC	Mice	Male	miR-21	AntagomiR	I.V.	Yes	Angiogenesis	Detrimental
Yuan et al., 2017a [47]	IRI	Mice	Female	miR-223	MSCs	I.V.	Yes	Inflammation	miR suppression: Detrimental
Yuan et al., 2017b [48]	IRI	Rats	Male	miR-423-5p	Mimic, antagomiR	I.V.	Yes	ER, oxidative stress	Mimic: Detrimental; AntagomiR: Protective
Chen et al., 2016a [49]	IRI	Mice	Male	miR-16	Mimic, antagomiR	Left renal artery	Yes	Apoptosis	Mimic: Detrimental; AntagomiR: Protective

Study	AKI model	Species	Sex	miRNA	Type of Intervention	Route of administration	SCr, BUN or Injury Scores	Pathway Category	Intervention Outcome
Chen et al., 2016b [50]	Nephrotoxic	Mice	Male	miR-192-5p	AntagomiR	I.V.	Yes	Apoptosis	Protective
Dai et al., 2016[51]	IRI + LPS PC	Mice	Male	miR-146a	AntagomiR	I.V.	Yes	Inflammation	With LPS PC: Detrimental Without LPS PC: Neutral
Gu et al., 2016 [52]	IRI	Rats	Male	miR-30	EVs	I.V.	Yes	Apoptosis	Protective
Vinas et al., 2016 [53]	IRI	Mice	Male	miR-486-5p	EVs	I.V.	Yes	Apoptosis	Protective
Wei et al., 2016 [54]	IRI	Mice	Male	miR-489	AntagomiR	I.V.	Yes	Apoptosis	Detrimental
Bhatt et al., 2015 [55]	IRI	Mice	NS	miR-687	AntagomiR	I.V.	Yes	Apoptosis, cell cycle activation	Protective
Collino et al., 2015 [56]	Rhabdo-myolysis	Mice	Male	Multiple	MSCs, EVs	I.V.	Yes	Multiple	Detrimental
Jia et al., 2015 [57]	Sepsis + Xe PC	Mice	Male	miR-21	AntagomiR	I.P.	Yes	Apoptosis, inflammation	Detrimental
Liang et al., 2015 [58]	IRI	Mice	NS	miR-26a	Mimic	I.V.	Yes	Inflammation	Protective
Liu et al., 2015 [59]	IRI	Rats	Male	miR-21	AntagomiR	I.P.	Yes	Autophagy	Protective
Ranganathan et al., 2015 [60]	IRI	Mice	NS	miR-150	Gene deletion	Gene deletion	Yes	Apoptosis	Protective
Bijkerk et al., 2014 [61]	IRI	Mice	NS	miR-126	BM cells	I.V.	Yes	Vasculo-genesis	Protective
Li et al., 2014 [62]	Candidemia	Mice	NS	miR-204 and miR-211	Mimic, antagomiR	I.V.	Yes	Unclear	miR-204 and miR-211 mimic: Protective; AntagomiR: Detrimental

Study	AKI model	Species	Sex	miRNA	Type of Intervention	Route of administration	SCr, BUN or Injury Scores	Pathway Category	Intervention Outcome
Lorenzen et al., 2014 [63]	IRI	Mice	Male	miR-24	AntagomiR	I.P.	Yes	Apoptosis	Protective
Pellegrini et al., 2014 [64]	Nephrotoxic	Mice	Both	miR-155	Gene deletion	Gene deletion	Yes	Apoptosis	Detrimental
Jia et al., 2013 [65]	IRI + Xe PC	Mice	Male	miR-21	AntagomiR	I.P.	Yes	Apoptosis	Detrimental
Cantaluppi et al., 2012 [66]	IRI	Rats	Male	miR-126 and miR-296	MVs	I.V.	Yes	Angiogenesis, tissue repair	Protective
Lan et al., 2012 [67]	IRI	Mice	Male	miR-494	Mimic, antagomiR	Intra-arterial	Yes	Apoptosis, inflammation	Mimic: Detrimental; AntagomiR: Protective
Reis et al., 2012 [68]	Nephrotoxic	Rats	Female	Multiple	BMSCs, CM	I.V.	Yes	Unclear	Protective
Xu et al., 2012 [69]	IRI + IPC	Mice	Male	miR-21	AntagomiR	I.V.	Yes	Apoptosis	With IPC: Detrimental Without IPC: Neutral
Wei et al., 2010 [70]	IRI	Mice	Male	Multiple	Gene deletion	Gene deletion	Yes	Multiple	Protective

Abbreviations: AKI, acute kidney injury; BM, bone marrow; BMSC, bone marrow mesenchymal stromal cell; BUN, blood urea nitrogen; CLP, cecal ligation and puncture; CM, conditioned media; DHCA, deep hypothermic circulatory arrest; ER, endoplasmic reticulum; EV, extracellular vesicles; I.P. intraperitoneal; IPC, ischemic pre-conditioning; IRI, ischemia-reperfusion injury; I.V., intravenous; LPS, lipopolysaccharide; MRSA, methicillin-resistant staphylococcus aureus; MSC, mesenchymal stem cells; MV, microvesicle; NS, not specified; PC, pre-conditioning; S.C., subcutaneous; SCr, serum creatinine; Tg, transgenic; Xe, Xenon.

Supplementary References

1 Du, T. *et al.* Microvesicles derived from human umbilical cord mesenchymal stem cells ameliorate renal ischemia-reperfusion injury via delivery of miR-21. *Cell Cycle* **19**, 1285-1297 (2020).

2 Geng, X. *et al.* LncRNA GAS5 promotes apoptosis as a competing endogenous RNA for miR-21 via thrombospondin 1 in ischemic AKI. *Cell Death Discovery* **6**, 19; https://doi.org/10.1038/s41420-020-0253-8 (2020).

3 Wang, X. *et al.* miR-218 Expressed in Endothelial Progenitor Cells Contributes to the Development and Repair of the Kidney Microvasculature. *The American Journal of Pathology* **190**, 642-659 (2020).

4 Wei, W., Yao, Y. Y., Bi, H. Y., Zhai, Z. & Gao, Y. miR-21 protects against lipopolysaccharide-stimulated acute kidney injury and apoptosis by targeting CDK6. *Ann. Transl. Med.* **8**, 303 (2020).

5 Yan, Y. *et al.* miR-214 represses mitofusin-2 to promote renal tubular apoptosis in ischemic acute kidney injury. *American Journal of Physiology Renal Physiology* **318**, F878-F887 (2020).

6 Yan, Z., Zang, B., Gong, X., Ren, J. & Wang, R. MiR-214-3p exacerbates kidney damages and inflammation induced by hyperlipidemic pancreatitis complicated with acute renal injury. *Life Sci.* **241**, 117118; https://doi.org/10.1016/j.lfs.2019.117118 (2020).

7 Zhao, X. R. *et al.* MicroRNA-27a-3p aggravates renal ischemia/reperfusion injury by promoting oxidative stress via targeting growth factor receptor-bound protein 2. *Pharmacol. Res.* **155**, 104718; https://doi.org/10.1016/j.phrs.2020.104718 (2020).

8 Chen, S. *et al.* MiR-424 is over-expressed and attenuates ischemia-reperfusion kidney injury via p53 and death receptor 6 pathway. *American Journal of Translational Research* **11**, 1965-1979; https://doi.org/ (2019).

9 Funahashi, Y. *et al.* miR-146a targeted to splenic macrophages prevents sepsis-induced multiple organ injury. *Lab. Invest.* **99**, 1130-1142 (2019).

10 Li, Z. L. *et al.* HIF-1alpha inducing exosomal microRNA-23a expression mediates the cross-talk between tubular epithelial cells and macrophages in tubulointerstitial inflammation. *Kidney Int.* **95**, 388-404 (2019).

11 Liu, Z. *et al.* The protective effect of miR-377 inhibitor against renal ischemia-reperfusion injury through inhibition of inflammation and oxidative stress via a VEGF-dependent mechanism in mice. *Mol. Immunol.* **106**, 153-158 (2019).

12 Ma, J., Li, Y. T., Zhang, S. X., Fu, S. Z. & Ye, X. Z. MiR-590-3p Attenuates Acute Kidney Injury by Inhibiting Tumor Necrosis Factor Receptor-Associated Factor 6 in Septic Mice. *Inflammation* **42**, 637-649 (2019).

13 Pan, T. *et al.* Delayed Remote Ischemic Preconditioning ConfersRenoprotection against Septic Acute Kidney Injury via Exosomal miR-21. *Theranostics* **9**, 405-423 (2019).

14 Qin, Y., Wang, G. & Peng, Z. MicroRNA-191-5p diminished sepsis-induced acute kidney injury through targeting oxidative stress responsive 1 in rat models. *Biosciences Reports* **39**, BSR20190548; https://doi.org/10.1042/BSR20190548 (2019).

15 Tapparo, M. *et al.* Renal Regenerative Potential of Extracellular Vesicles Derived from miRNA-Engineered Mesenchymal Stromal Cells. *Int. J. Mol. Sci.* **20**, 2381; https://doi.org/10.3390/ijms20102381 (2019).
16 Wu, X. Q. *et al.* miR-191 secreted by platelet-derived microvesicles induced apoptosis of renal tubular epithelial cells and participated in renal ischemia-reperfusion injury via inhibiting CBS. *Cell Cycle* **18**, 119-129 (2019).
17 Yue, J. *et al.* MicroRNA-187 Reduces Acute Ischemic Renal Podocyte Injury via Targeting Acetylcholinesterase. *J. Surg. Res.* **244**, 302-311 (2019).
18 Zhang, W., Chen, C., Jing, R., Liu, T. & Liu, B. Remote Ischemic Preconditioning Protects Cisplatin-Induced Acute Kidney Injury through the PTEN/AKT Signaling Pathway. *Oxid. Med. Cell. Longev.* **2019**, 7629396; https://doi.org/10.1155/2019/7629396 (2019).
19 Zhang, X. B. *et al.* Inhibition of miR-155 Ameliorates Acute Kidney Injury by Apoptosis Involving the Regulation on TCF4/Wnt/beta-Catenin Pathway. *Nephron* **143**, 135-147 (2019).
20 Zhang, C. *et al.* miR-30c-5p Reduces Renal Ischemia-Reperfusion Involving Macrophage. *Med. Sci. Monit.* **25**, 4362-4369 (2019).
21 Zhu, Y., Yin, X., Li, J. & Zhang, L. Overexpression of microRNA-204-5p alleviates renal ischemia-reperfusion injury in mice through blockage of Fas/FasL pathway. *Exp. Cell Res.* **381**, 208-214 (2019).
22 Chen, S. *et al.* Micro RNA-155 inhibitor as a potential therapeutic strategy for the treatment of acute kidney injury (AKI): a nanomedicine perspective. *RSC Advances* **8**, 15890-15896 (2018).
23 Guo, Y. *et al.* MicroRNA-709 Mediates Acute Tubular Injury through Effects on Mitochondrial Function. *J. Am. Soc. Nephrol.* **29**, 449-461 (2018).
24 Li, H., Ma, Y., Chen, B. & Shi, J. miR-182 enhances acute kidney injury by promoting apoptosis involving the targeting and regulation of TCF7L2/Wnt/beta-catenins pathway. *Eur. J. Pharmacol.* **831**, 20-27 (2018).
25 Li, X. Y., Zhang, Y. Q., Xu, G., Li, S. H. & Li, H. miR-124/MCP-1 signaling pathway modulates the protective effect of itraconazole on acute kidney injury in a mouse model of disseminated candidiasis. *Int. J. Mol. Med.* **41**, 3468-3476; https://doi.org/10.3892/ijmm.2018.3564 (2018).
26 Liu, X. Y. *et al.* Renal inhibition of miR-181a ameliorates 5-fluorouracil-induced mesangial cell apoptosis and nephrotoxicity. *Cell Death and Disease* **9**, 610; https://doi.org/10.1038/s41419-018-0677-8 (2018).
27 Mathia, S. *et al.* A dual role of miR-22 in rhabdomyolysis-induced acute kidney injury. *Acta Physiologica (Oxf)* **224**, e13102; https://doi.org/10.1111/apha.13102 (2018).
28 Song, N. *et al.* miR-21 Protects Against Ischemia/Reperfusion-Induced Acute Kidney Injury by Preventing Epithelial Cell Apoptosis and Inhibiting Dendritic Cell Maturation. *Front. Physiol.* **9**, 790; https://doi.org/10.3389/fphys.2018.00790 (2018).
29 Song, T. *et al.* miR-17-92 ameliorates renal ischemia reperfusion injury. *The Kaohsiung Journal of Medical Sciences* **34**, 263-273 (2018).

30 Wei, Q. *et al.* MicroRNA-668 represses MTP18 to preserve mitochondrial dynamics in ischemic acute kidney injury. *J. Clin. Invest.* **128**, 5448-5464 (2018).
31 Yu, L., Gu, T., Liu, Y., Jiang, X. & Shi, E. Overexpression of MicroRNA-106b-5p Attenuates Kidney Injuries after Deep Hypothermic Circulatory Arrest in Rats. *The Journal of Thoracic and Cardiovascular Surgery* **66**, 109-115 (2018).
32 Zhu, X., Li, W. & Li, H. miR-214 ameliorates acute kidney injury via targeting DKK3 and activating of Wnt/beta-catenin signaling pathway. *Biol. Res.* **51**, 31; https://doi.org/10.1186/s40659-018-0179-2 (2018).
33 Amrouche, L. *et al.* MicroRNA-146a in Human and Experimental Ischemic AKI: CXCL8-Dependent Mechanism of Action. *J. Am. Soc. Nephrol.* **28**, 479-493 (2017).
34 Colbert, J. F. *et al.* A model-specific role of microRNA-223 as a mediator of kidney injury during experimental sepsis. *American Journal of Physiology Renal Physiology* **313**, F553-F559 (2017).
35 de Almeida, D. C. *et al.* A Regulatory miRNA-mRNA Network Is Associated with Tissue Repair Induced by Mesenchymal Stromal Cells in Acute Kidney Injury. *Front. Immunol.* **7**, 645; https://doi.org/10.3389/fimmu.2016.00645 (2016).
36 Fu, D. *et al.* MiRNA-21 has effects to protect kidney injury induced by sepsis. *Biomedicine and Pharmacotheraphy* **94**, 1138-1144 (2017).
37 Hao, J. *et al.* Induction of microRNA-17-5p by p53 protects against renal ischemia-reperfusion injury by targeting death receptor 6. *Kidney Int.* **91**, 106-118 (2017).
38 Huang, Y. *et al.* Regulation and mechanism of miR-146 on renal ischemia reperfusion injury. *Pharmazie* **73**, 29-34 (2018).
39 Jia, P. *et al.* MicroRNA-21 Is Required for Local and Remote Ischemic Preconditioning in Multiple Organ Protection Against Sepsis. *Crit. Care Med.* **45**, e703-e710; https://doi.org/10.1097/CCM.0000000000002363 (2017).
40 Jiao, X. *et al.* miR-21 contributes to renal protection by targeting prolyl hydroxylase domain protein 2 in delayed ischaemic preconditioning. *Nephrology (Carlton)* **22**, 366-373 (2017).
41 Ranghino, A. *et al.* The effects of glomerular and tubular renal progenitors and derived extracellular vesicles on recovery from acute kidney injury. *Stem Cell Research and Therapy* **8**, 24; https://doi.org/10.1186/s13287-017-0478-5 (2017).
42 Wang, J. *et al.* MBD2 upregulates miR-301a-5p to induce kidney cell apoptosis during vancomycin-induced AKI. *Cell Death Discovery* **8**, e3120; https://doi.org/10.1038/cddis.2017.509 (2017).
43 Wang, S., Zhang, Z., Wang, J. & Miao, H. MiR-107 induces TNF-alpha secretion in endothelial cells causing tubular cell injury in patients with septic acute kidney injury. *Biochem. Biophys. Res. Commun.* **483**, 45-51 (2017).
44 Wilflingseder, J. *et al.* miR-182-5p Inhibition Ameliorates Ischemic Acute Kidney Injury. *Am. J. Pathol.* **187**, 70-79 (2017).
45 Xu, X. *et al.* Role of miR21 on vascular endothelial cells in the protective effect of renal delayed ischemic preconditioning. *Mol. Med. Report.* **16**, 2627-2635 (2017).
46 Xu, X. *et al.* Renal Protection Mediated by Hypoxia Inducible Factor-1alpha Depends on Proangiogenesis Function of miR-21 by Targeting Thrombospondin 1. *Transplantation* **101**, 1811-1819 (2017).

47 Yuan, X., Wang, X., Chen, C., Zhou, J. & Han, M. Bone mesenchymal stem cells ameliorate ischemia/reperfusion-induced damage in renal epithelial cells via microRNA-223. *Stem Cell Research and Therapy* **8**, 146; https://doi.org/10.1186/s13287-017-0599-x (2017).
48 Yuan, X. P. *et al.* MicroRNA-423-5p facilitates hypoxia/reoxygenation-induced apoptosis in renal proximal tubular epithelial cells by targeting GSTM1 via endoplasmic reticulum stress. *Oncotarget* **8**, 82064-82077 (2017).
49 Chen, H. H. *et al.* Urinary miR-16 transactivated by C/EBPbeta reduces kidney function after ischemia/reperfusion-induced injury. *Sci. Rep.* **6**, 27945; https://doi.org/10.1038/srep27945 (2016).
50 Chen, J. *et al.* p53 activates miR-192-5p to mediate vancomycin induced AKI. *Sci. Rep.* **6**, 38868; https://doi.org/10.1038/srep38868 (2016).
51 Dai, Y. *et al.* miR-146a is essential for lipopolysaccharide (LPS)-induced cross-tolerance against kidney ischemia/reperfusion injury in mice. *Sci. Rep.* **6**, 27091; https://doi.org/10.1038/srep27091 (2016).
52 Gu, D. *et al.* Mesenchymal Stromal Cells Derived Extracellular Vesicles Ameliorate Acute Renal Ischemia Reperfusion Injury by Inhibition of Mitochondrial Fission through miR-30. *Stem Cells Int.* **2016**, 2093940 (2016).
53 Vinas, J. L. *et al.* Transfer of microRNA-486-5p from human endothelial colony forming cell-derived exosomes reduces ischemic kidney injury. *Kidney Int.* **90**, 1238-1250 (2016).
54 Wei, Q. *et al.* MicroRNA-489 Induction by Hypoxia-Inducible Factor-1 Protects against Ischemic Kidney Injury. *J. Am. Soc. Nephrol.* **27**, 2784-2796 (2016).
55 Bhatt, K. *et al.* MicroRNA-687 Induced by Hypoxia-Inducible Factor-1 Targets Phosphatase and Tensin Homolog in Renal Ischemia-Reperfusion Injury. *J. Am. Soc. Nephrol.* **26**, 1588-1596 (2015).
56 Collino, F. *et al.* AKI Recovery Induced by Mesenchymal Stromal Cell-Derived Extracellular Vesicles Carrying MicroRNAs. *J. Am. Soc. Nephrol.* **26**, 2349-2360 (2015).
57 Jia, P. *et al.* Xenon Protects Against Septic Acute Kidney Injury via miR-21 Target Signaling Pathway. *Crit. Care Med.* **43**, e250-e259; https://doi.org/10.1097/CCM.0000000000001001 (2015).
58 Liang, S., Wang, W. & Gou, X. MicroRNA 26a modulates regulatory T cells expansion and attenuates renal ischemia-reperfusion injury. *Mol. Immunol.* **65**, 321-327 (2015).
59 Liu, X. *et al.* MiR-21 inhibits autophagy by targeting Rab11a in renal ischemia/reperfusion. *Exp. Cell Res.* **338**, 64-69 (2015).
60 Ranganathan, P. *et al.* MicroRNA-150 deletion in mice protects kidney from myocardial infarction-induced acute kidney injury. *American Journal of Physiology Renal Physiology* **309**, F551-F558 (2015).
61 Bijkerk, R. *et al.* Hematopoietic microRNA-126 protects against renal ischemia/reperfusion injury by promoting vascular integrity. *J. Am. Soc. Nephrol.* **25**, 1710-1722 (2014).
62 Li, X. Y., Zhang, K., Jiang, Z. Y. & Cai, L. H. MiR-204/miR-211 downregulation contributes to candidemia-induced kidney injuries via derepression of Hmx1 expression. *Life Sci.* **102**, 139-144 (2014).

63 Lorenzen, J. M. *et al.* MicroRNA-24 antagonism prevents renal ischemia reperfusion injury. *J. Am. Soc. Nephrol.* **25**, 2717-2729 (2014).
64 Pellegrini, K. L. *et al.* MicroRNA-155 deficient mice experience heightened kidney toxicity when dosed with cisplatin. *Toxicol. Sci.* **141**, 484-492 (2014).
65 Jia, P. *et al.* miR-21 contributes to xenon-conferred amelioration of renal ischemia-reperfusion injury in mice. *Anesthesiology* **119**, 621-630 (2013).
66 Cantaluppi, V. *et al.* Microvesicles derived from endothelial progenitor cells protect the kidney from ischemia-reperfusion injury by microRNA-dependent reprogramming of resident renal cells. *Kidney Int.* **82**, 412-427 (2012).
67 Lan, Y. F. *et al.* MicroRNA-494 reduces ATF3 expression and promotes AKI. *J. Am. Soc. Nephrol.* **23**, 2012-2023 (2012).
68 Reis, L. A. *et al.* Bone marrow-derived mesenchymal stem cells repaired but did not prevent gentamicin-induced acute kidney injury through paracrine effects in rats. *PLoS One* **7**, e44092; https://doi.org/10.1371/journal.pone.0044092 (2012).
69 Xu, X. *et al.* Delayed ischemic preconditioning contributes to renal protection by upregulation of miR-21. *Kidney Int.* **82**, 1167-1175 (2012).
70 Wei, Q. *et al.* Targeted deletion of Dicer from proximal tubules protects against renal ischemia-reperfusion injury. *J. Am. Soc. Nephrol.* **21**, 756-761 (2010).

Supplementary Table S2. Quality of Evidence (GRADE Method) for serum creatinine and injury score outcomes in studies used in the meta-analyses.

Intervention	Study design	Risk of Bias	Inconsistency	Indirectness	Imprecision	Publication bias	Upgrading factors	Quality of evidence
**miRNA-21 (serum creatinine)**	High quality	Serious (-1)	Very serious (-2)	Serious (-1)	Serious (-1)	Uncertain	None	Very low quality (+)
**miRNA-21 (injury scores)**	High quality	Serious (-1)	Not an issue	Serious (-1)	Serious (-1)	Uncertain	None	Very low quality (+)

Supplementary Table S3. PRISMA 2009 Checklist

Section/topic	#	Checklist item	Reported on page #
**TITLE**			
Title	1	Identify the report as a systematic review, meta-analysis, or both.	1
**ABSTRACT**			
Structured summary	2	Provide a structured summary including, as applicable: background; objectives; data sources; study eligibility criteria, participants, and interventions; study appraisal and synthesis methods; results; limitations; conclusions and implications of key findings; systematic review registration number.	2
**INTRODUCTION**			
Rationale	3	Describe the rationale for the review in the context of what is already known.	3
Objectives	4	Provide an explicit statement of questions being addressed with reference to participants, interventions, comparisons, outcomes, and study design (PICOS).	3
**METHODS**			
Protocol and registration	5	Indicate if a review protocol exists, if and where it can be accessed (e.g., Web address), and, if available, provide registration information including registration number.	12
Eligibility criteria	6	Specify study characteristics (e.g., PICOS, length of follow-up) and report characteristics (e.g., years considered, language, publication status) used as criteria for eligibility, giving rationale.	12-13
Information sources	7	Describe all information sources (e.g., databases with dates of coverage, contact with study authors to identify additional studies) in the search and date last searched.	12-13
Search	8	Present full electronic search strategy for at least one database, including any limits used, such that it could be repeated.	Supplementary file 1
Study selection	9	State the process for selecting studies (i.e., screening, eligibility, included in systematic review, and, if applicable, included in the meta-analysis).	13-14
Data collection process	10	Describe method of data extraction from reports (e.g., piloted forms, independently, in duplicate) and any processes for obtaining and confirming data from investigators.	13-14
Data items	11	List and define all variables for which data were sought (e.g., PICOS, funding sources) and any assumptions and simplifications made.	13-14,20
Risk of bias in individual studies	12	Describe methods used for assessing risk of bias of individual studies (including specification of whether this was done at the study or outcome level), and how this information is to be used in any data synthesis.	8, 14
Summary measures	13	State the principal summary measures (e.g., risk ratio, difference in means).	14
Synthesis of results	14	Describe the methods of handling data and combining results of studies, if done, including measures of consistency (e.g., I^2) for each meta-analysis.	14-15

Section/topic	#	Checklist item	Reported on page #
Risk of bias across studies	15	Specify any assessment of risk of bias that may affect the cumulative evidence (e.g., publication bias, selective reporting within studies).	9, Fig. 6
Additional analyses	16	Describe methods of additional analyses (e.g., sensitivity or subgroup analyses, meta-regression), if done, indicating which were pre-specified.	14-15
**RESULTS**			
Study selection	17	Give numbers of studies screened, assessed for eligibility, and included in the review, with reasons for exclusions at each stage, ideally with a flow diagram.	4, Fig.1
Study characteristics	18	For each study, present characteristics for which data were extracted (e.g., study size, PICOS, follow-up period) and provide the citations.	4-5, Fig. 2, S1
Risk of bias within studies	19	Present data on risk of bias of each study and, if available, any outcome level assessment (see item 12).	8, Fig. 6
Results of individual studies	20	For all outcomes considered (benefits or harms), present, for each study: (a) simple summary data for each intervention group (b) effect estimates and confidence intervals, ideally with a forest plot.	4-7, Table 1, Fig. 2, Fig. 4, Fig. 5, S1
Synthesis of results	21	Present results of each meta-analysis done, including confidence intervals and measures of consistency.	6-8, Fig. 4-5
Risk of bias across studies	22	Present results of any assessment of risk of bias across studies (see Item 15).	8, Fig. 6
Additional analysis	23	Give results of additional analyses, if done (e.g., sensitivity or subgroup analyses, meta-regression [see Item 16]).	5, Table 1
**DISCUSSION**			
Summary of evidence	24	Summarize the main findings including the strength of evidence for each main outcome; consider their relevance to key groups (e.g., healthcare providers, users, and policy makers).	9-12
Limitations	25	Discuss limitations at study and outcome level (e.g., risk of bias), and at review-level (e.g., incomplete retrieval of identified research, reporting bias).	11-12
Conclusions	26	Provide a general interpretation of the results in the context of other evidence, and implications for future research.	9-12
**FUNDING**			
Funding	27	Describe sources of funding for the systematic review and other support (e.g., supply of data); role of funders for the systematic review.	20

SUPPLEMENTARY FILE 1. Search Strategy

Search strategy. <1946 to April 30, 2020.
Database: Embase Classic+Embase <1947 to 2020 April 30>, Ovid MEDLINE(R) ALL <1946 to April 30, 2020>

----

1 exp MicroRNAs/ (186072)
2 (MicroRNA* or Micro RNA* or non coding rna* or noncoding rna* or Small Temporal rna* or miRNA* or mir).tw,kw. (245314)
3 mir.kf. (11880)
4 (microparticle* or ectosome* or exosome* or microvesicle* or extracellular vesicle*).tw,kw. (68208)
5 cell-derived microparticles/ or exosomes/ (26167)
6 or/1-5 (320418)
7 exp Acute Kidney Injury/ (118688)
8 (acute adj2 (renal or kidney)).tw. (115644)
9 ((acute or ischem* or ischaem* or reperfusion) and (kidney or renal)).kf. (8221)
10 ((ischem* or ischaem* or reperfusion) adj3 (renal or kidney)).tw. (21732)
11 aki.tw,kw. (26688)
12 ((acute or toxic or contrast induced) adj2 nephropath*).tw. (6320)
13 ((acute or toxic or contrast induced) and nephropath*).kf. (520)
14 nephrotoxi*.tw,kw. (53599)
15 or/7-14 (225622)
16 6 and 15 (1243)
17 limit 16 to yr="1990 -Current" (1236)
18 17 use medall (465)
19 exp microRNA/ (186072)
20 (MicroRNA* or Micro RNA* or non coding rna* or Small Temporal rna* or miRNA* or mir).tw. (228445)
21 exosome/ (17725)
22 membrane microparticle/ (4096)
23 (microparticle* or ectosome* or exosome* or microvesicle* or extracellular vesicle*).tw. (66231)
24 19 or 20 or 21 or 22 or 23 (303849)
25 exp Acute Kidney Injury/ (118688)
26 (acute adj2 (renal or kidney)).tw. (115644)
27 aki.tw. (26352)
28 kidney ischemia/ (10463)
29 contrast induced nephropathy/ (4241)
30 ((acute or toxic or contrast induced) adj2 nephropath*).tw. (6320)
31 ((ischem* or ischaem* or reperfusion) adj3 (renal or kidney)).tw. (21732)
32 nephrotoxicity/ (60934)
33 or/25-32 (238340)
34 24 and 33 (1161)
35 limit 34 to yr="1990 -Current" (1156)
36 35 use emczd (798)

37 18 or 36 (1263)
38 remove duplicates from 37 (882)
39 38 use medall (461) Medline
40 38 use emczd (421) Embase

Web of Science – up to April 30, 2020
